# Botanicals to Control Soft Rot Bacteria of Potato

**DOI:** 10.1100/2012/796472

**Published:** 2012-05-22

**Authors:** M. M. Rahman, A. A. Khan, M. E. Ali, I. H. Mian, A. M. Akanda, S. B. Abd Hamid

**Affiliations:** ^1^Department of Plant Pathology, Bangabadhu Sheikh Mujibur Rahman Agricultural University, Gazipur 1706, Bangladesh; ^2^Center for Research in Nanotechnology and Catalysis, University of Malaya, 50603 Kuala Lumpuor, Malaysia

## Abstract

Extracts from eleven different plant species such as jute (*Corchorus capsularis* L.), cheerota (*Swertia chiraita* Ham.), chatim (*Alstonia scholaris* L.), mander (*Erythrina variegata*), bael (*Aegle marmelos* L.), marigold (*Tagetes erecta*), onion (*Allium cepa*), garlic (*Allium sativum* L.), neem (*Azadiracta indica*), lime (*Citrus aurantifolia*), and turmeric (*Curcuma longa* L.) were tested for antibacterial activity against potato soft rot bacteria, *E. carotovora* subsp. *carotovora (Ecc)* P-138, under *in vitro* and storage conditions. Previously, *Ecc* P-138 was identified as the most aggressive soft rot bacterium in Bangladeshi potatoes. Of the 11 different plant extracts, only extracts from dried jute leaves and cheerota significantly inhibited growth of *Ecc* P-138 *in vitro*. Finally, both plant extracts were tested to control the soft rot disease of potato tuber under storage conditions. In a 22-week storage condition, the treated potatoes were significantly more protected against the soft rot infection than those of untreated samples in terms of infection rate and weight loss. The jute leaf extracts showed more pronounced inhibitory effects on *Ecc*-138 growth both in *in vitro* and storage experiments.

## 1. Introduction

Indiscriminate use of chemical pesticides to control various pests and pathogenic microorganisms of crops plants is causing health hazard both in terrestrial and aquatic lives through their residual toxicity [1, 2]. Considering the adverse and alarming effects of synthetic pesticides on environment and natural habitats, this study was undertaken to find out an alternative and nontoxic biological control agents [3] to control the soft rot bacterial pathogens in Bangladeshi potatoes. Green plants are a huge reservoir of various effective chemotherapeutics and could serve as an environmentally friendly natural alternative to the toxic chemical pesticides [4].

During the recent decades, many herbal extracts have been extensively tested and a myriad of reports have been documented outlining the uses of plant extracts to control the animal and plant diseases [5–7]. A good number of reports outlined the antimicrobial effects of some medicinal plants for plant disease control [7]. Some plant extracts were documented as effective inhibitors of phytopathogenic bacteria [5, 6]. Antimicrobial activities of several plant extracts against bacterial soft rot of potatoes were evaluated and a quite satisfactory result was obtained [8, 9]. The liquid extract of hemp flowers and essential oils were tested against *Ecc*, the causal bacterium of potato soft rot, and satisfactory results were documented [8]. However, no attempts have been made to identify and characterize the antibacterial plant extracts to control the soft rot bacterial pathogens of potatoes in Bangladesh. In this paper, we investigated the anti-Ecc P-138 activity of 11 different plant extracts and documented antibacterial activity in jute leaf and cheerota extracts in *in vitro* and storage experiments.

## 2. Materials and Methods

### 2.1. Selection of Plants and Preparation of Extracts

A total of 11 plants, namely, jute (*Corchorus capsularis* L.), cheerota (*Swertia chirata* Ham.), chatim (*Alstonia scholaris* L.), mander (*Erythrina variegata*), bael (*Aegle marmelos* L.), marigold (*Tagete serecta*), onion (*Allium cepa*), garlic (*Allium sativum* L.), neem (*Azadirachta indica*), lime (*Citrus aurantifolia*), and turmeric (*Curcuma longa* L.) were tested in this investigation (Table 1). Dried jute leaves, whole plant of cheerota, bark of chatim and mandar were used for the preparation of extracts at the ratio of 1 : 10 (w/v) in water. Plant parts were soaked or submerged in distilled water for 20–24  h. Water was chosen as an extraction media because of its low cost, easier availability, and biocompatibility. The water extracts were collected by passing through double-layered muslin cloth at least two times. To prepare extracts of other plants, different plant parts like leaves, roots, bulbs, and rhizomes were crushed in a mortar and pestle. The crushed materials were mixed with distilled water at 1 : 1 (w/v) and blended in an electrical blender. They were filtered through double layered muslin cloth at least two times. The extracts were poured into conical flasks and used as stock solution. Mouth of each flask was closed with aluminum foil and preserved in a refrigerator at 4°C for future uses. 

### 2.2. Bioassay of Plant Extracts against Soft Rot Bacteria

Antibacterial activity of each plant extracts (Table 1) was tested against *Ecc *P-138, the most virulent soft rot bacterial strain of Bangladeshi potatoes, through the growth inhibition test *in vitro *[8, 10]. *Ecc *P-138 (10^8^ cfu/ml) was inoculated on autoclaved YPDA media at 28°C for 24 h to obtain pure culture of *Ecc* P-138. A fresh YPDA medium was then amended with 30, 50, 75 and 90% plant extracts and was autoclaved. The medium was poured into petri dishes at the rate of 20 ml/dish. After solidification, the amended medium was spot inoculated with the pure culture of *Ecc *P-138. The spot inoculated plates were incubated in an incubator at 30°C for 14 days and the bacterial growth was recorded to determine the inhibitory effects of the plant extracts. The plates were arranged in the incubator following complete randomized design with three replications (petri dishes).

### 2.3. Effect of Jute Leaf and Cheerota Extracts on Soft Rot Disease of Storage Potatoes

Based on encouraging results of the bioassay, extracts of dry jute leaf and cheerota plant were selected to evaluate their efficacy to protect potatoes against soft rot bacteria under storage conditions. Two extracts were prepared in distilled water at a ratio of 1 : 10 (v/v) following the procedures as described above. Seven hundred grams of potato tubers were treated with each of the plant extracts. The potato tuber bulbs were submerged in the extracts for 30 minutes and air-dried at room temperature. Inocula of the soft rot bacteria, *Ecc* P-138 were prepared at a concentration of 10^8^ cfu mL^−1^ following the same procedures as described under *in vitro* test. Plant extract treated potato tubers were inoculated with the inocula of *Ecc *P-138. For inoculation, the inoculum suspensions were sprayed over the tubers uniformly using an automizer. Inoculated tubers were air-dried and stored at room temperature. An uninoculated control was maintained for each variety. Visual observations were made after 2, 6, 10, 14, 18, and 22 weeks of inoculation and data on the number of soft rot infected tubers was recorded and loss in weight due to soft rot in storage was computed and expressed in percentage (w/w) using the formula [11] given below:

(1) $$\begin{array}{c} \text{Infection}\;\text{\%}=\frac{\text{No}.\;\text{of}\;\text{infected}\;\text{tubers}}{\text{Total}\;\text{no}.\;\text{of}\;\text{tubers}}\times 100,\\ \text{Loss}\;\text{of}\;\text{weight}\;\text{\%}=\frac{\text{Initial}\;\text{weight}-\text{weight}\;\text{after}\;\text{discarding}\;\text{the}\;\text{infected}\;\text{sample}}{\text{Initial}\;\text{weight}}\times 100. \end{array}$$

Percentage of disease reduction (PDR) was calculated following formula shown below [12]:

(2) $$\begin{array}{c} \text{PDR}=\frac{\text{Ack}-\text{Atr}}{\text{Ack}}\times 100, \end{array}$$

where, Ack is disease severity/loss (by weight) in control and Atr is disease severity/loss (by weight) in treatment.

## 3. Results

### 3.1. Antibacterial Assay of Plant Extracts *In Vitro*

**Figure 1:**
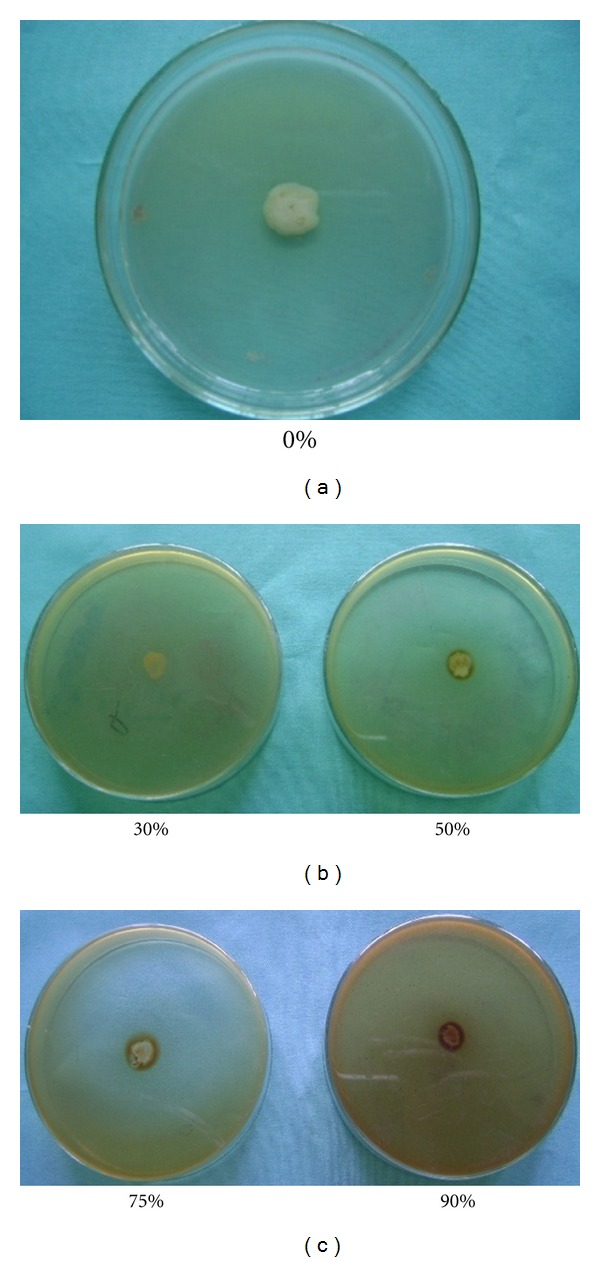
Antibacterial activity of jute leaf extract against *Ecc* P-138 at different concentrations of the extract in YPDA medium.

**Figure 2:**
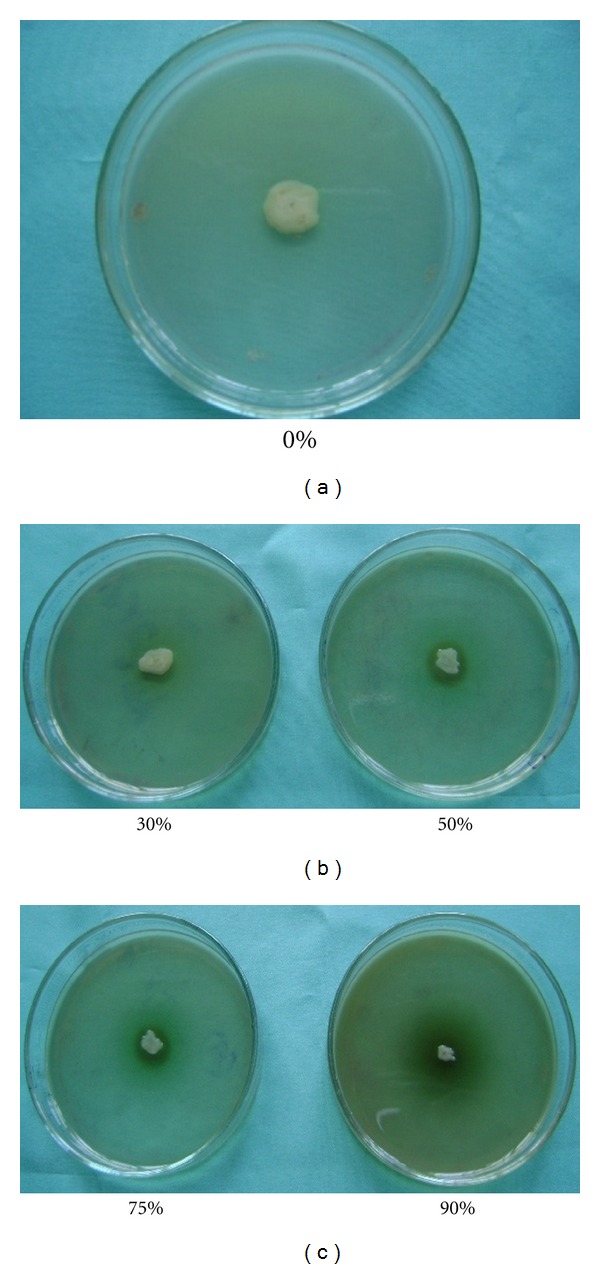
Antibacterial activity of cheerota extract against *Ecc * P-138 at different concentrations of extract in YPDA medium.

Out of 11 different plant extracts, only extracts of dry jute leaf and cheerota suppressed the growth of the soft rot bacteria, *Ecc* P-138, in 50–90% extracts containing YPDA medium (Table 2). This was confirmed with the visual appearance of inhibition zones around the soft rot bacterium *Ecc* P-138 (Figures 1 and 2). Higher antibacterial activity of the extracts was observed at higher concentration. This was reflected by the higher thickness of the inhibition zones around the soft rot bacterial strain. The jute leaf extract demonstrated more inhibition than that of the cheerota against potato soft rot *Ecc* P-138 in triplicate experiments. On the basis of *in vitro* test, jute leaf and cheerota plant extracts were selected for treatment of potato tubers against soft rot disease under storage. Other nine plant extracts did not show antibacterial activity (Table 3). 

### 3.2. Effect of Jute Leaf and Cheerota Extracts on Potato Tubers under Storage

**Figure 3:**
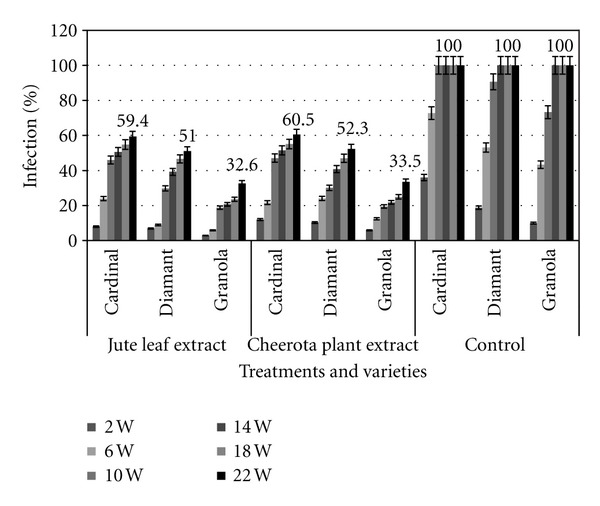
Effect of tuber treatment with jute leaf extract and cheerota plant extract on soft rot disease incidence of potato in storage.

**Figure 4:**
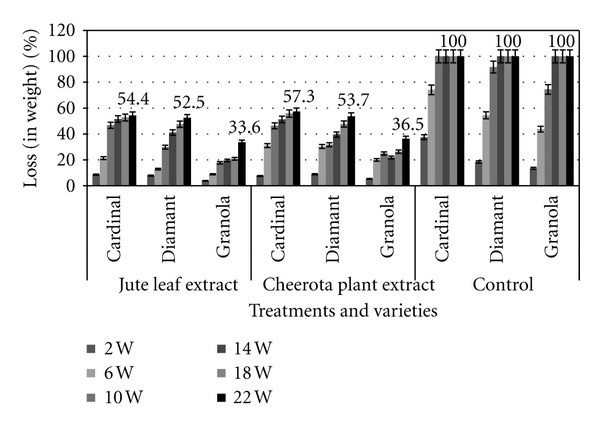
Effect of tuber treatment with jute leaf extract and cheerota plant extract on percentage of loss in weight of potato in storage condition.

**Figure 5:**
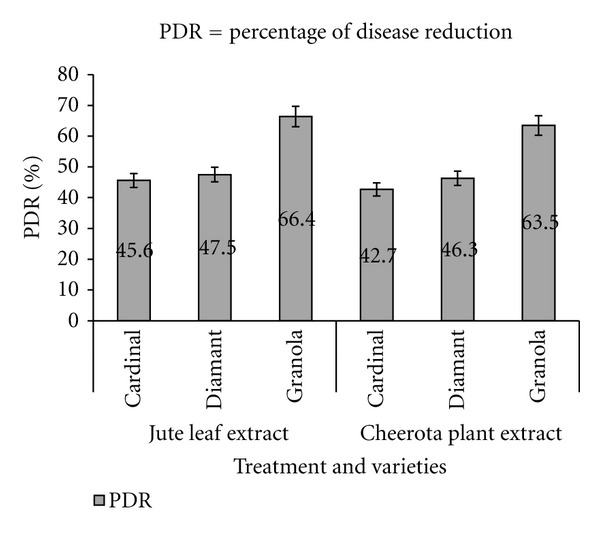
Effect of two botanical extracts on percentage of disease reduction (PDR) of potato after 22 weeks of inoculation in storage condition.

The rates of soft rot infection and tuber damage in treated and untreated potatoes are demonstrated in Figures 3 and 4, respectively. In case of untreated tubers, the infection rate was much higher and 100% potato tubers were damaged within 14 weeks of storage. On the other hand, the infection rate and damage were significantly lowered (20–50%) in treated samples (Figures 3 and 4). Approximately ~40–70% of treated samples survived even in 22 weeks of storage. The maximum infection rate and damage were observed in the cardinal and diamante varieties and the lowest infection rate and damage were found in granola varieties. The cardinal and diamante varieties of potatoes also demonstrated the highest incidence of disease and the granola varieties showed lowest incidence of that in the jute leaf and cheerota-plant-extract-treated samples after 22 weeks of storage (Figure 5). 

## 4. Discussions

The use of herbal extracts to control plant diseases is an environment friendly approach and an effective alternative to toxic chemical pesticides. Krebs and Jaggir [8] investigated a water extract of hemp flowers and essential oils against *E. carotovora* causal agent of potato soft rot. The extracts were tested *in vitro* using a pure bacterial culture and *in vivo* on potatoes latently infected with the pathogen. The protective effect was most pronounced with the extract of hemp flowers. Since very few botanical extracts have been documented to control the soft rot pathogens and many works reported the antibacterial action of herbal extracts against a good number of bacterial pathogens [7, 13, 14], the identification of effective plant extracts against the potato soft rot bacterium, *Ecc *P-138, was undertaken to control the potato soft rot diseases in Bangladesh. 

In this study, the effects of plant extracts and their efficiencies against bacterial soft rot of potatoes were evaluated *in vitro* and in storage conditions, and quite satisfactory results were obtained with jute leaf and cheerota extracts. The inhibitory activity of plant extracts was most likely due to antimicrobial components present in plant extracts. However, the exact chemical compounds and their controlling mechanism to the soft rot bacteria need to be elucidated. Since jute is the first economic plant in Bangladesh, the jute leaves are readily available without any cost. Additionally, the decomposition of jute leaves further increases the soil fertility. Thus the use of jute leaf extracts should not have any phytotoxic effects on other plants. However, its effects on epiphytic beneficial microorganisms need to be addressed before making any recommendation. 

## 5. Conclusion

Jute leaf (dried) (*Corchorus capsularis *L.) and cheerota plant (*Swertia chirata *Ham.) extracts showed antibacterial activity against soft rot bacteria *Ecc *P-138 *in vitro* and effectively reduced the bacterial soft rot disease of different potato varieties in storage conditions. Since jute leaves are readily available without any cost in Bangladesh, the application of dry jute leaf extracts is a viable alternative to toxic chemical pesticides to control the soft rot diseases in Bangladeshi potatoes. 

## Figures and Tables

**Table 1:** List of plants tested to control bacterial soft rot pathogens of potato.

Name of plants		Family	Parts used
English/Bangla	Scientific name		
Jute	*Corchorus capsularis *L.	Tiliaceae	Dry leaves
Cheerota	*Swertia chirata *Ham.	Gentianaceae	Whole plant
Devils tree	*Alstonias cholaris *L.	Apocynaceae	Bark
Coral tree	*Erythrina indica*	Leguminosae	Bark
Bael	*Aegle marmelos *L.	Rutaceae	Young fruits and leaves
Marigold	*Tagetes serecta*	Compositae	Leaves and roots
Onion	*Allium cepa*	Lilliaceae	Bulbs and leaves
Garlic	*Allium sativum *L.	Lilliaceae	Bulbs and leaves
Neem	*Azadirachta indica*	Meliaceae	Leaves
Lime	*Citrus aurantifolia*	Rutaceae	Leaves
Turmeric	*Curcuma longa *L.	Zingiberaceae	Rhizome

**Table 2:** *In vitro* evaluation of antibacterial activity of dried jute leaf extract and cheerota plant extract against potato soft rot bacteria (*E. carotovora *subsp*. Carotovora *P-138).

Concentration of plant extract (%)	Jute leaf extract *E. carotovora *subsp*. carotovora* (P-138)
30	−
50	−
75	++
90	++++
Control*	− −
	Cheerota plant extract
30	−
50	−
75	++
90	+++
Control*	− −

−/− −: did not show antibacterial activity; ++: medium antibacterial activity; +++/++++: strong antibacterial activity; − −: good growth of soft rot bacteria; *: plant extract was not added in medium.

**Table 3:** *In vitro* evaluation of antibacterial activity of nineplant extracts against soft rot bacteria of potato.

Treatments with plant extracts (low-to-high concentration)	Antibacterial activity against soft rot bacteria at different time of intervals
	*E. carotovora* subsp*. carotovora* (P-138)
*Aegle marmelos *L.	−
*Alstonia scholaris *L.	−
*Erythrina variegata*	−
*Tagetes erecta*	−
*Allium cepa*	−
*Allium sativum L.*	−
*Azadirachta indica*	−
*Citrus aurantifolia*	−
*Curcuma longa L.*	−
Control*	− −

−/− −: no antibacterial activity showed; − −: good growth of soft rot bacteria; *: no treatment with plant extracts.
